# Divergence within and among Seaweed Siblings (*Fucus vesiculosus* and *F*. *radicans*) in the Baltic Sea

**DOI:** 10.1371/journal.pone.0161266

**Published:** 2016-08-15

**Authors:** Angelica Ardehed, Daniel Johansson, Lisa Sundqvist, Ellen Schagerström, Zuzanna Zagrodzka, Nikolaj A. Kovaltchouk, Lena Bergström, Lena Kautsky, Marina Rafajlovic, Ricardo T. Pereyra, Kerstin Johannesson

**Affiliations:** 1 Department of Biological and Environmental Sciences, University of Gothenburg, Gothenburg, Sweden; 2 Centre for Marine Evolutionary Biology, University of Gothenburg, Gothenburg, Sweden; 3 Department of Marine Sciences, University of Gothenburg, Gothenburg, Sweden; 4 Department of Ecology, Environment and Plant Sciences, Stockholm University, Stockholm, Sweden; 5 Department of Marine Sciences at Tjärnö, University of Gothenburg, Strömstad. Sweden; 6 Komarov Botanical Institute of Russian Academy of Sciences, St Petersburg, Russia; 7 Department of Aquatic Resources, Swedish University of Agricultural Sciences, Öregrund, Sweden; 8 Department of Physics, University of Gothenburg, Gothenburg, Sweden; Chinese Academy of Sciences, CHINA

## Abstract

Closely related taxa provide significant case studies for understanding evolution of new species but may simultaneously challenge species identification and definition. In the Baltic Sea, two dominant and perennial brown algae share a very recent ancestry. *Fucus vesiculosus* invaded this recently formed postglacial sea 8000 years ago and shortly thereafter *Fucus radicans* diverged from this lineage as an endemic species. In the Baltic Sea both species reproduce sexually but also recruit fully fertile new individuals by asexual fragmentation. Earlier studies have shown local differences in morphology and genetics between the two taxa in the northern and western Bothnian Sea, and around the island of Saaremaa in Estonia, but geographic patterns seem in conflict with a single origin of *F*. *radicans*. To investigate the relationship between northern and Estonian distributions, we analysed the genetic variation using 9 microsatellite loci in populations from eastern Bothnian Sea, Archipelago Sea and the Gulf of Finland. These populations are located in between earlier studied populations. However, instead of bridging the disparate genetic gap between N-W Bothnian Sea and Estonia, as expected from a simple isolation-by-distance model, the new populations substantially increased overall genetic diversity and showed to be strongly divergent from the two earlier analysed regions, showing signs of additional distinct populations. Contrasting earlier findings of increased asexual recruitment in low salinity in the Bothnian Sea, we found high levels of sexual reproduction in some of the Gulf of Finland populations that inhabit extremely low salinity. The new data generated in this study supports the earlier conclusion of two reproductively isolated but very closely related species. However, the new results also add considerable genetic and morphological complexity within species. This makes species separation at geographic scales more demanding and suggests a need for more comprehensive approaches to further disentangle the intriguing relationship and history of the Baltic Sea fucoids.

## Introduction

The Biological Species Concept defines species as reproductively isolated units [1], and current gene flow is consequently a key parameter to understand the relationship among closely related taxa. Somewhat simplified, individuals of a species should be able to exchange genes while individuals of different species should not. However, geographic isolation, local adaptation, and extrinsic barriers to gene flow hamper gene flow within species. For this reason, sometimes population genetic divergence within a species may be of a similar order of magnitude as genetic divergence between closely related species [2]. High sharing of ancestral genetic variation due to a recent common ancestry, or introgression and hybridization, will further complicate discrimination of young species [3–5].

Contrasting the general marine paradigm of efficient dispersal of propagules, many macroalgal species show clear patterns of isolation by distance and strong geographic population genetic structures [6]. This is not least true for species of fucoid brown algae [7], including species inhabiting the Baltic Sea [8, 9]. In particular, two of these species, *Fucus vesiculosus* L. and *Fucus radicans* Bergström & Kautsky, share a very recent (<8000 years) common ancestry [9] resulting in poor separation in barcoding genes [10], and sharing of most microsatellite alleles [11]. Despite a close genetic relationship, experimental studies have unveiled differences in both physiological and ecological traits between the two species [12–15]. This suggests niche separation that may promote their co-existence [16].

As a rare feature among fucoid species, Baltic Sea populations of *F*. *vesiculosus* and *F*. *radicans* may, in addition to sexual recruitment, recruit new attached and fully fertile individuals asexually, by release of small fragments (adventitious branches) [17]. Notably, asexual recruitment has generated a few extensively distributed and very old clones present in the N-W Bothnian Sea, while in more southern Baltic populations including *F*. *radicans* in Estonia, sexual recruitment is almost exclusive [18, 19].

*Fucus vesiculosus* and *F*. *radicans* overlap in distribution in large parts of the northern Baltic Sea. Careful analyses using microsatellite genetic variation have repeatedly shown that, at a local scale, barriers to gene flow separate the two species [9–11]. This is true both in N-W Bothnian Sea and in Estonia (island of Saaremaa). However, species-separation at a geographic scale is more complex, as illustrated by the comparison of the Estonian and N-W Bothnian Sea populations. Here, isolation-by-distance effects within species result in a primary division of all populations into two geographic groups (one for Estonia and one for N-W Bothnian Sea), and thereafter a secondary division into *F*. *radicans* and *F*. *vesiculosus* within each geographic cluster [9].

The main aim of this study was to further investigate the reason for the geographic subdivision into two separate geographic clusters. We hypothesized that if isolation-by-distance causes this division, populations located between the N-W Bothnian Sea and Estonia would show an intermediate position in a genetic analysis. Thus we analysed genetic variation in populations from, the coasts of E Bothnian Sea, Archipelago Sea and Gulf of Finland and integrated the new data with earlier data from N-W Bothnian Sea and Estonia [9, 11, 18, 19]. In addition, we included two "outlier population", one from the Baltic Proper, and one from the North Sea. We used the same nine microsatellite loci and the same analytical approaches as used earlier [18]. Genetic variation has prior to this study not been analysed in populations from E Bothnian Sea, Archipelago Sea and Gulf of Finland, but early morphological studies (before the discovery of *F*. *radicans* as a separate species, [10]) have shown a large range of morphotypes of *Fucus "vesiculosus"* inside Gulf of Finland [20, 21].

According to an earlier suggested hypothesis, high level of asexual recruitment of populations of *Fucus vesiculosus* and *F*. *radicans* in the northern Baltic Sea is due to extremely low ambient salinity in the Bothnian Sea [18, 19]. This is based on reports of negative effects on egg-sperm interactions [22]. In the present study we also tested the "low salinity"-hypothesis by examining the degree of asexually recruitment in *Fucus* in the Gulf of Finland where *Fucus* is present in very low salinities towards the east end of the gulf.

Our results largely supported the earlier conclusion of two locally distinct species, although in part of the Gulf of Finland we failed to assign individuals to species based on morphological criteria. Populations providing a geographic link between the Estonia and N-W Bothnian Sea populations did not appear as genetic intermediates. Instead the new populations added to the complexity of the genetic structure observed in each species. Finally, the low-salinity effect on asexual recruitment was not supported in the Gulf of Finland where some populations remained highly sexual despite extremely low salinities.

## Materials and Methods

### The Baltic Sea environment

The Baltic Sea is one of the largest brackish water bodies in the world. It has a young and complex postglacial history with passing through various stages of brackish- and freshwater during the past 15,000 years. Its current status as a large brackish water sea was established as recently as 8000 years ago [23, 24]. Currently, salinities range from 2 practical salinity units (PSU) in the north, to about 20–25 PSU at the entrance to the North Sea NE of Denmark [25] with the steepest salinity gradients in the Danish straits. The low salinity presents a challenge to many marine organisms, and several species in the Baltic Sea live close to their physiological limits [26, 27]. A general trend is also that the populations of the Baltic Sea are genetically differentiated from, and have lower genetic diversity than populations of the same species living outside the Baltic Sea [28].

### Study sites and sampling

We sampled 20–60 thalli (occasionally fewer) of either *F*. *radicans* or *F*. *vesiculosus*, or when possible both, from 12 populations in the northern and eastern parts of the Baltic Sea that has not been genetically analysed earlier. The sampling of this species did not require permission from any local or national authority as sampled only in areas with full access to anyone to pick seaweeds. The sampled species are not classified as endangered, and are not under any protection in the sampled area. The same sampling strategy was used for the new samples as for the old ones included in the population genetic analyses. That is, in each site, individual ramets were sampled either along a transect of up to 100 m, or from within an area of 50-200m x 50–200 m. Sampling ramets grown from the same (or close) holdfasts (<1m distance) were strictly avoided. From morphology we assigned each sampled individual to either *F*. *vesiculosus* or *F*. *radicans*, using thallus width and overall appearance, following the species description [10]. However, thalli from four of these 12 populations (R, S, T, U) from the Finnish coast of Gulf of Finland, were left unassigned as the morphology of these thalli were intermediate to morphologies of *F*. *vesiculosus* and *F*. *radicans* from other areas. The morphological identification was done prior to genotyping of all individuals.

Additional genetic data from 18 populations, earlier analysed for genetic variation using the same microsatellite loci as for the new populations (see below), were included in this study. These data were from previously published studies [18, 19]. Ten of the total of 30 populations sampled were from sites where the two species were sampled in the same spot (small scale sympatry) and these populations are denoted D1/D2, E1/E2, F1/F2, V1/V2, W1/W2 with the first populations being *F*. *radicans* and the second from the same site being *F*. *vesiculosus* (Table 1). Despite an extended period of sampling (2003–2010), all samples have been treated in the same way and genetic analyses have been performed in the same laboratory using the exact same methods (described in [18, 19]). Altogether, genetic information from 1101 individual *Fucus* thalli were included in the analyses.

**Table 1:** Populations of *Fucus* included in this study.

Sampling site	Pop.	Coordinates	Region	Species	Ramets (n)	Genets (MLGs)	Sampled
Kristineberg	A	58°15' N, 11°27' E	North Sea	*F*. *vesiculosus*	42	42	2003#
Öland	B	57°21' N, 17°03' E	Baltic Proper	*F*. *vesiculosus*	43	42	2003#
Öregrund	C	60°22' N, 18°21' E	W Bothnian Sea	*F*. *radicans*	48	13	2003#
Djursten	D1	60°23' N, 18°24' E	W Bothnian Sea	*F*. *radicans*	48	16	2007#
Djursten	D2	60°23' N, 18°24' E	W Bothnian Sea	*F*. *vesiculosus*	51	32	2007#
Gävle	E1	60°49' N, 17°17' E	W Bothnian Sea	*F*. *radicans*	34	3	2010§
Gävle	E2	60°49' N, 17°17' E	W Bothnian Sea	*F*. *vesiculosus*	26	14	2010§
Bönhamn	F1	62°53' N, 18°18' E	N Bothnian Sea	*F*. *radicans*	30	5	2007#
Bönhamn	F2	62°53' N, 18°18' E	N Bothnian Sea	*F*. *vesiculosus*	34	23	2007#
Skagsudde	G	63°11' N, 19°01' E	N Bothnian Sea	*F*. *radicans*	47	9	2010&
Drivan	H	63°26' N, 19°20' E	N Bothnian Sea	*F*. *radicans*	60	7	2010&
Järnäs	I	63°26' N, 19°39' E	N Bothnian Sea	*F*. *radicans*	59	8	2010&
Hällkalla	J	63°25' N, 20°57' E	N Bothnian Sea	*F*. *radicans*	50	12	2007#
South Vallgrund	K	63°09' N, 21°13' E	N Bothnian Sea	*F*. *radicans*	50	5	2007#
Märigrund	L	62°31' N, 21°03' E	N Bothnian Sea	*F*. *radicans*	45	33	2007#
Sälskär	M	62°19' N, 21°10' E	N Bothnian Sea	*F*. *radicans*	29	29	2007#
Kaskö	N	62°20' N, 21°12' E	N Bothnian Sea	*F*. *vesiculosus*	20	9	2010§
Lankoori	O	61°26' N, 21°27' E	E Bothnian Sea	*F*. *vesiculosus*	26	19	2010§
Santakaari	P	61°06' N, 21°17' E	E Bothnian Sea	*F*. *vesiculosus*	50	50	2010§
Nurmesanuokka	Q	61°11' N, 21°20' E	Archipelago Sea	*F*. *vesiculosus*	50	50	2010§
Kotka Rankki	R	60°22' N, 26°57' E	Gulf of Finland	Unassigned	23	23	2005§
Kotka Kirkonmaa	S	60°22' N, 27°02' E	Gulf of Finland	Unassigned	18	12	2005§
Virolahti Suurpisi	T	60°26' N, 27°38' E	Gulf of Finland	Unassigned	40	38	2005§
Virolahti Parrio	U	60°27' N, 27°38' E	Gulf of Finland	Unassigned	26	23	2005§
Seskar Island	V1	60°00' N, 28°38' E	Gulf of Finland	*F*. *radicans*	40	31	2010§
Seskar Island	V2	60°00' N, 28°38' E	Gulf of Finland	*F*. *vesiculosus*	40	23	2010§
Pulli panki	W1	58°36' N, 22°58' E	Estonia	*F*. *radicans*	15	15	2006#
Pulli panki	W2	58°36' N, 22°58' E	Estonia	*F*. *vesiculosus*	8	8	2006#
Triigi	X	58°35' N, 22°43' E	Estonia	*F*. *radicans*	25	25	2006#
Köiguste	Y	58°22' N, 22°58' E	Estonia	*F*. *vesiculosus*	24	23	2006#

#) Data from Johannesson et al 2011

§) New samples

&) Data from Ardehed et al 2015

### DNA extraction and microsatellite genotyping

Genomic DNA was extracted using the NucleoSpin® Plant II-kit (MACHEREY-NAGEL, Düren, Germany) according to standard kit protocol. DNA samples were amplified in a thermal cycler following the same procedure as [18], and genotyped at nine microsatellite loci (L20, L38, L58, L85, L94 [29]; and Fsp1, Fsp2, Fsp3, Fsp4 [30]). PCR products were pool-plexed and sized on a Beckman-Coulter CEQ 8000 capillary sequencer, and fragments were analysed using the Fragment Analysis Software (Beckman-Coulter Inc., Fullerton, CA, USA).

### Statistical analysis

The microsatellite data sets from the 12 new populations were checked for errors such as null alleles, stuttering, and large allelic drop-out, by means of 1000 randomizations using the software MICRO-CHECKER v. 2.2.3 [31]. For clones, this was done at genet level, that is, one copy of each multi locus genotype (MLG) was checked. The software GENEPOP 4.2 [32] was used to estimate allele frequencies, and deviations from Hardy-Weinberg equilibrium within populations (*F*_IS_) and for each locus separately following Weir and Cockerham [33] and with corrections for multiple testing according to [34]. Tests of linkage disequilibrium (*LD*) for each pair of loci were also performed separately on each population. We also calculated between-population divergence using pair-wise estimates of *F*_ST_ obtained on genet level from GENEPOP between populations of each species but we allowed the four unassigned populations to be included in both matrixes.

### Population genetic structure

The program GENCLONE 2.0 [35] was used for recognition of the number of ramets (replicate MLGs/thalli of the same clone) and genets (distinct MLGs/thalli of different genotypes) within each population. From this we determined the number of individuals belonging to clones and the number of unique MLGs (“singletons”) present in each site.

To describe the population genetic structure among all populations we applied several different methods in order to test the robustness of our result under different assumptions (of the different methods). We used a conservative approach in that we performed the population-level analyses on data including only the genets of each population. To test if removing all ramets except one copy would make a difference to the results, we rerun several of the tests on data including all ramets. As the results were indifferent to analyses including only genets, we present here only results based on genets. The principal component analysis (PCA) estimates the genetic relationships among populations in a multidimensional space, depicting the genetic relatedness among populations regardless of historical or other causes underlying this structure [36]. Using the software PCAgen 1.2.1 [37], this relationship was projected on the first two dimensions as a "genetic map". Furthermore we used a Bayesian approach, implemented in the software STRUCTURE 2.3.3 [38] to assess population differentiation both with and without prior information of sample information (locality), and allowing for admixture. This program identifies populations by assigning individuals over K populations minimizing deviation from Hardy-Weinberg and is based on the assumption of populations being in Hardy Weinberg-equilibrium. We used the STRUCTURE analysis to approach two issues, the first being the genetic structure of the populations along the eastern coasts of the Baltic Sea, from north Finland, over the Gulf of Finland to Estonia, spanning the northern Baltic and the Estonian populations and the genetic gap between these areas described in earlier studies [9]. The second issue was to assess the local genetic divergence between *F*. *vesiculosus* and *F*. *radicans* individuals living in sympatric populations. For the latter analysis we used a subset of five local pairs of sympatric populations, each pair was sampled at the same coordinates. In all STRUCTURE analyses, preliminary simulations were executed to determine burn-in length and full run lengths. For the final analyses, three repeat simulations at each K-value were used, with a burn-in period of 10,000 and a run length of 100,000 generations.

Finally, we applied a new approach that groups populations according to the directional relative migration between populations [39]. This approach provides network plots that visualise patterns of genetic structure. With this analysis we focused on disentangling the relationship between the populations analysed for the first time in this study, and nearby populations on either side of these, thus including all Finnish, Russian and Estonian populations. All calculations were performed using the web application divMigrate-online using D [40] as a measure of genetic differentiation [39].

## Results

Analyses from MICRO-CHECKER on data from the 12 new populations (genotype copies excluded) showed no evidence of null alleles, except occasionally in some populations for the locus Fsp2. With low null allele frequency (0.20) and expected minor effects on detection of genetic differentiation [41], this locus was maintained for subsequent analyses. There was no evidence of scoring errors from large allelic dropout or stuttering at any loci in the 12 data sets. No pairs of microsatellite loci were significantly linked across samples (Bonferroni corrected tests on genets only).

The nine scored loci were all polymorphic in the 30 populations. A total of 122 alleles were identified and the average number of alleles per locus was 13.4, ranging from 5 (*L*85) to 30 (*Fsp*2). Only taking into account genet variation, seven of the nine loci showed populations with departures from Hardy-Weinberg equilibrium (S1 Table). In four of the seven loci these included few departures, or a balance between heterozygote excess and deficiency, but three loci (Fsp2, Fsp3 and L20) stood out as having a higher number of populations with heterozygote deficiency than populations with heterozygote excess. Overall, 44 cases of heterozygote deficiency was found compared to 16 cases of heterozygote excess. The deviations from Hardy-Weinberg were rather evenly distributed among populations, and most populations showed no or a low a balanced number of deficiencies and excesses. We found, however, a tendency of heterozygote deficiency in all the Estonian populations (W1, W2, X, Y) with 15 cases of heterozygote deficiency and no case of heterozygote excess (S1 Table).

**Fig 1:**
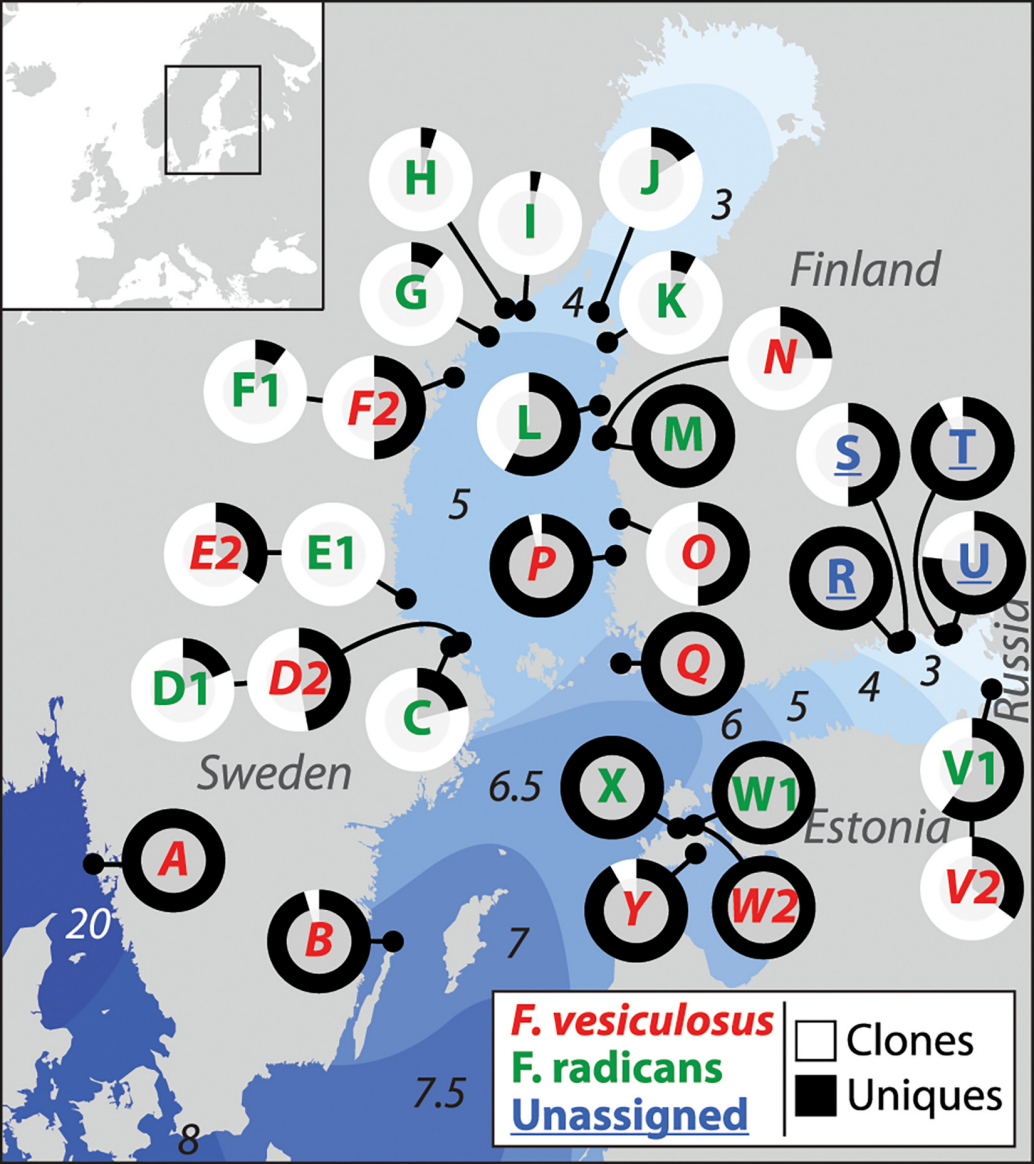
Proportions of unique and clonal ramets in Baltic Sea *Fucus vesiculosus* and *F*. *radicans* populations. Unique genotypes are sexually recruited or rare clones. Clonal ramets are all asexually recruited. Salinities are indicated in Practical Salinity Units. *F*. *vesiculosus* populations are marked in red and italic letters, *F*. *radicans* in green and regular letters, and four unassigned populations (see text) are marked in blue and underlined letters. Populations of the two species having the same letters are sympatric.

Clonal richness (ratio: genets/ramets) varied greatly among populations (Table 1). In low salinity there was a trend towards a strong dominance of clones in populations of N-W Bothnian Sea, but in the innermost parts of the Gulf of Finland three of five populations remained largely sexually recruited despite very low salinities (Fig 1).

**Fig 2:**
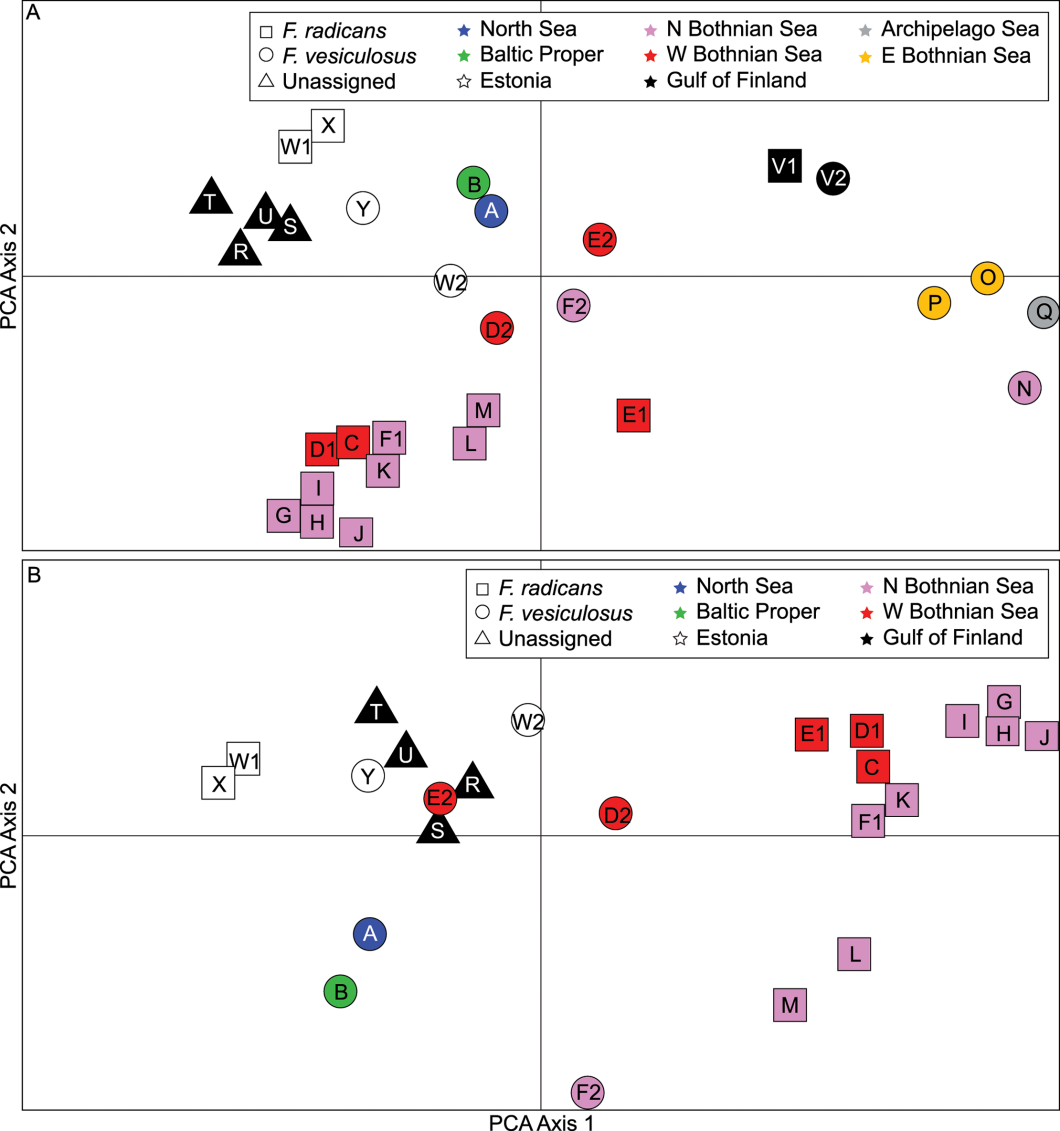
Principal component analysis separating populations based on microsatellite allele frequency distributions. (A) All populations analysed together, including 12 of *F*. *vesiculosus* (circles), 13 of *F*. *radicans* (squares), and 4 unassigned (triangles). Colours indicate different regions (**blue** = North Sea; **green** = Baltic Proper; **white** = Estonian coast; **purple** = north Bothnian Sea; **red** = west Bothnian Sea; **yellow** = east Bothnian Sea; **grey** = Archipelago Sea and **black** = Gulf of Finland). Populations with the same letter are sympatric. Analysis was done on genet level with 10,000 randomizations. The overall *F*_*ST*_ is 0.19, which indicates a highly significant genetic structure (*p* = 0.0001). (B) Same populations as in (A) but the more divergent populations (N, O, P, Q and V) removed to better resolve the relationship between the remaining populations.

**Fig 3:**
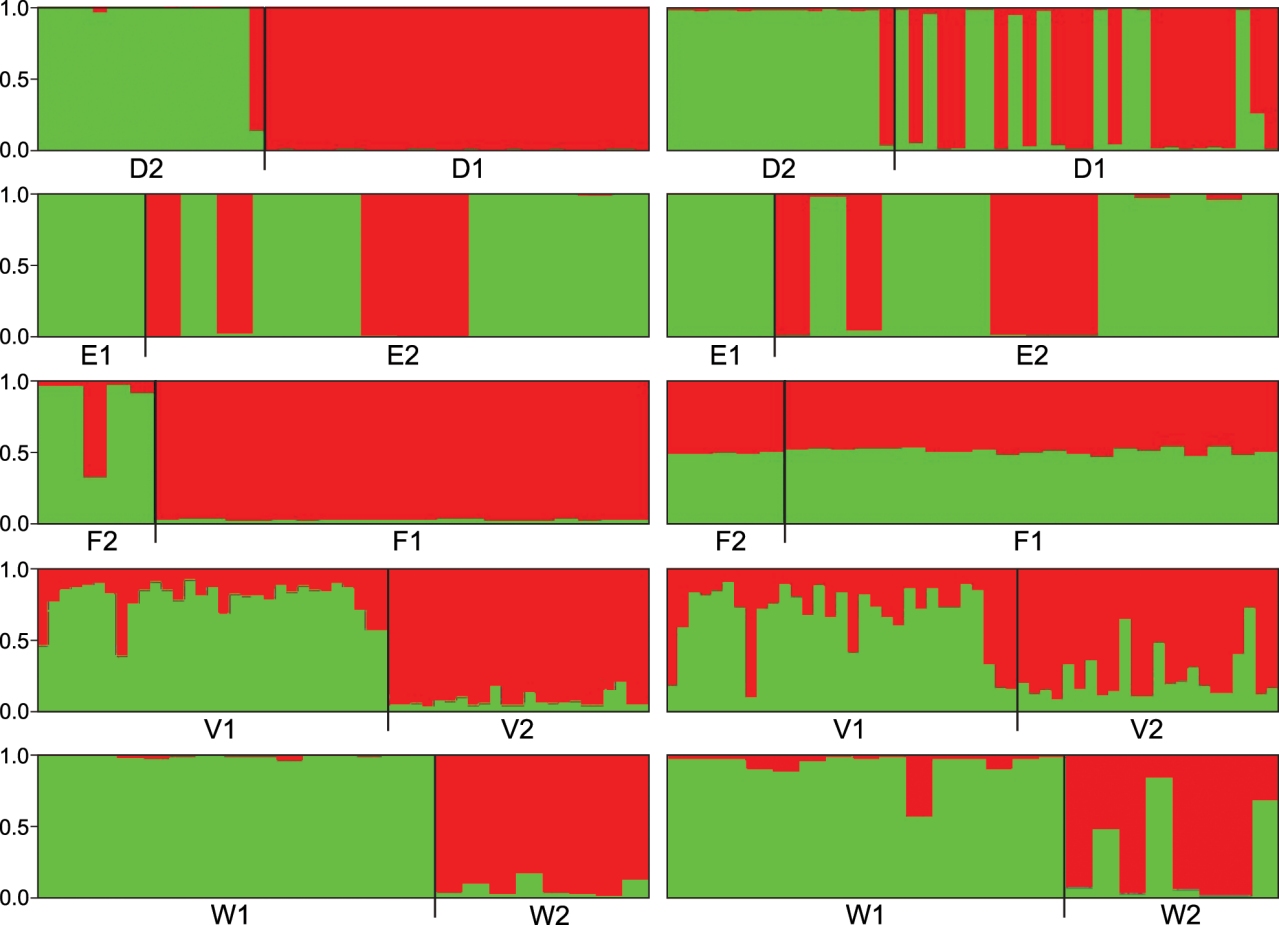
STRUCTURE analyses of five pairs of sympatric *Fucus* populations. Populations D1, E1, F1, V1 and W1 are *F*. *radicans* while D2, E2, F2, V2 and W2 are *F*. *vesiculosus*. Analyses are based on genetic variation in 9 microsatellite loci. Vertical bars indicate assignment probability for each genet to any of two genetic groups (K = 2). Left side of the panel are analyses with the software option LOCPRIOR, while right side are without LOCPRIOR.

Genetic differentiation within each species ranged widely with pairwise *F*_ST_ estimates from 0.0 to 0.35 in *F*. *radicans* and from 0.0 to 0.31 in *F*. *vesiculosus* (S2 Table). The PCA analysis displayed the relative position of the 30 populations in a multidimensional genetic space (Fig 2), with the first two principal components explaining 51.1% (26.6% and 24.5%, respectively) of the total genetic variation among populations. As expected, both the total differentiation and differentiation along each of the two first axes, were highly significant (global *F*_*ST*_ = 0.19, *p*<0.0001; PCA1 *F*_*ST*_ = 0.050, *p*<0.0001; PCA2 *F*_*ST*_ = 0.046, *p*<0.0001). Overall, the genetic map unveiled some intriguing patterns: Firstly, there was no strong global separation of the two species. Secondly, separation was obvious in all local sympatric populations, although weak in the Russian Gulf of Finland site (V1 and V2, Fig 2A, but see below and Fig 3).

Overall there was a strong geographic component in the separation of samples in the way that samples of the same species from the same area grouped in tight clusters. However, the degree of differentiation among clusters appeared unlinked to the actual geographic distance in many cases. For example, populations N (N Bothnian Sea), and Q (Archipelago Sea) of *F*. *vesiculosus* appeared distantly related to geographically nearby populations of the same species (F2 nearby to N; D2, Y and W2 nearby to Q), while instead N and Q were genetically similar (Fig 2A). One additional example was the strong genetic separation between the Gulf of Finland populations from Finland (the unassigned R, S, T, U) and the nearby Russian populations of both species (V1 and V2). In contrast, several populations appeared genetically close while geographically distant. For example, the North Sea population of *F*. *vesiculosus* (A) appeared closely related to *F*. *vesiculosus* populations from the Baltic Proper (B), Estonia (Y), and W Bothnian Sea (D2, E2), and the unassigned Gulf of Finland populations seemed genetically related to the Estonian populations of both species (Fig 2A).

As the PCA analysis of all 30 populations were heavily influenced by the four eastern Bothnian Sea populations of *F*. *vesiculosus* (N, O, P, Q) and to some extend also by the two Russian populations (V1 and V2), we did a second PCA analysis, excluding these six populations in order to resolve the separation among the remaining populations more clearly. This analysis showed a more obvious separation between the two species with all populations of *F*. *radicans* (except the two from Estonia) well separated from all the *F*. *vesiculosus* populations along the first PC axis that now explained 34.5% of the variation (Fig 2B). Notably, the Estonian *F*. *radicans* populations were also separated from all *F*. *vesiculosus* populations but appeared even more distant to the other *F*. *radicans* populations along the PC-1 axis (Fig 2B). Also in this analysis, the difference between *F*. *vesiculosus* from the North Sea (A) and the Baltic Proper (B) was minor compared to the separation among the *F*. *vesiculosus* populations inside the Baltic Sea. Finally, the unassigned populations now seemed to appear well within the *F*. *vesiculosus* cluster (Fig 2B), instead of as before associated with the Estonian *F*. *radicans* populations (Fig 2A).

With the Bayesian STRUCTURE analysis we first addressed the issue of local genetic barriers to gene flow between truly sympatric populations of *F*. *vesiculosus* and *F*. *radicans*. Using information of species grouping as an input to the analysis gave, as expected, a stronger support for separation of the two species (Fig 3A–3E), than when performing the analysis without this prior information (Fig 3F–3J). These results largely corroborated the PCA analysis, but the separation of two pairs (E1/E2 and F1/F2) was less convincing than in the PCA analysis. On the other hand, the separation of the Russian Gulf of Finland samples (V1/V2) was more distinct in the STRUCTURE analysis (Fig 3) than in the PCA (Fig 2), clearly illustrating the importance to use different genetic analyses to obtain a more comprehensive view of the genetic relationship among populations.

**Fig 4:**
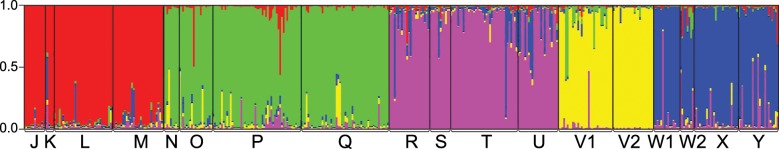
STRUCTURE analyses of Finnish, Russian and Estonian *Fucus* populations. Populations J, K, L, M, V1, W1 and X are *F*. *radicans* and N, O, P, Q, V2, W2 and Y are *Fucus vesiculosus*. Populations R, S, T and U were not possible to clearly assigned to species based on morphological criteria. All analyses are based on genetic variation in 9 microsatellite loci. Colour of vertical bars indicate individual genet assignment probabilities to any of five genetic groups (K = 5, which was supported by values of Pr (X|K, not shown). Black vertical lines separate the different populations.

The complex genetic structure along the Finnish, Russian and Estonian coasts, were further explored using a separate STRUCTURE analysis. This grouped populations of the same species and from the same local area in coherent clusters (Fig 4). As expected, population N that was identified as *F*. *vesiculosus* on thallus morphology, was correctly clustered with more southern populations of *F*. *vesiculosus* instead of with geographically nearby populations of *F*. *radicans* (Fig 4). This again supported a clear division in this area between the two species. However, the species division in the Gulf of Finland was much less clear. The four Finnish populations that could not be assigned to species based on the morphology prior to genetic analysis (R, S, T, U) remained separated from all other populations of both species. Furthermore, the Russian Gulf of Finland populations (V1 and V2) and the Estonian populations (W1, W2, X, Y) did not differentiate into species at the optimal level of structuring (K = 5) (Fig 4). Notably, the Finnish (R, S, T, U) and Russian (V1, V2) Gulf of Finland populations remained separated despite their relatively close geographic affinity (Fig 4).

**Fig 5:**
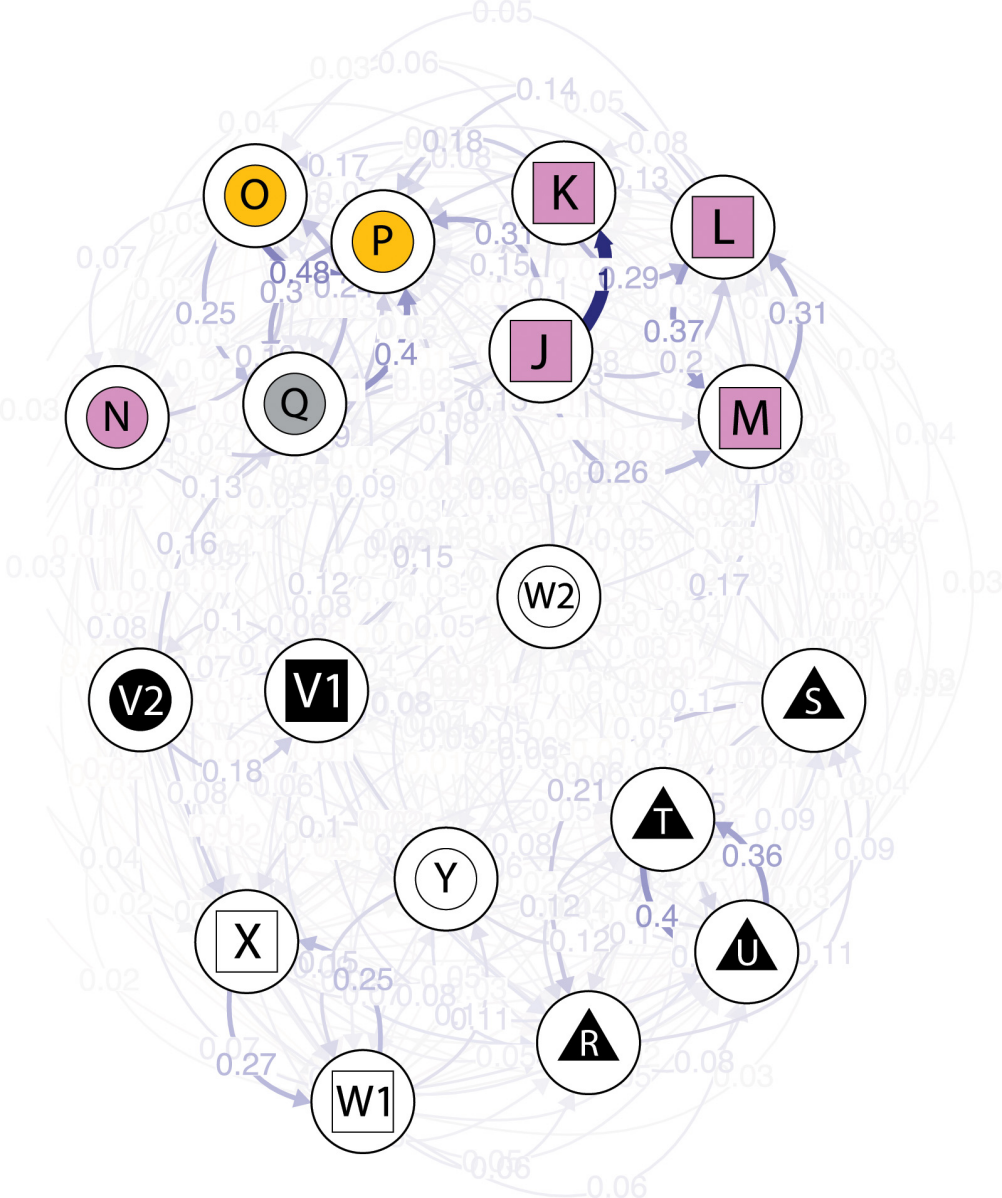
Directional relative migration network of Finnish, Russian and Estonian *Fucus* populations. Species and areas are designated the same symbols and colours as in Fig 2. Population positions indicate relatedness from the perspective of gene flow. Arrows indicate the direction of gene flow, and numbers (and arrow shading/thickness) show the values of directional migration relative to the highest value in the analysis (in this case from population J to population K).

The relative migration-based network plot (Fig 5) performed on the same subset of populations as the STRUCTURE analysis (Fig 4) largely corroborated the grouping of the PCA and STRUCTURE analyses. Three main groups formed among the populations along the Finnish and Estonian coasts; one northern group of *F*. *radicans* (J, K, L, M), one north to eastern Bothnian Sea group of *F*. *vesiculosus* (N, O, P) also including the population from the Archipelago Sea (Q), and one group of the unassigned Gulf of Finland populations (R, S, T, U) (Fig 5). The two Russian populations (V1—*F*. *radicans* and V2—*F*. *vesiculosus*) were neighbours, while the sympatric Estonian populations of *F*. *radicans* (W1) and *F*. *vesiculosus* (W2) were clearly distinct in this analysis (Fig 5).

## Discussion

With large populations and a very recent common ancestry (less than a few thousand generations ago), we would expect only weak impact of genetic drift and no large genetic differences in neutral (microsatellite) markers between *F*. *radicans* and *F*. *vesiculosus* [10, 11, 42, 43]. Nevertheless, careful examination of divergence in truly sympatric sites where thalli of the two species are found mixed within the same microhabitat indicate barriers to gene flow between taxa. This result corroborates earlier findings [9–11] and supports the interpretation of the two entities being separate species.

However, in both species populations from different geographic areas are in many cases strongly genetically distinct. For example, populations of *F*. *radicans* from the Bothnian Sea are very different from Estonian populations (Fig 2A; W1 and X versus all other *F*. *radicans* populations), as also earlier reported [9]. In *F*. *vesiculosus* there is a similarly strong subdivision between the four populations sampled along the SW Finnish coast and all other populations of this species analysed in the present study (Fig 2A; compare N-Q to the rest of *F*. *vesiculosus* populations). Indeed, all other populations of *F*. *vesiculosus*, except the Russian Gulf of Finland population, form a coherent genetic cluster.

The taxonomic status of the four Gulf of Finland populations (R- U), that could not be assigned using the morphological criteria that have been used to separate the two species [10], remained inconclusive even after addition of genetic information. In the PCA of all populations (Fig 2A) they appear most associated to Estonian *F*. *radicans* populations. When the SW Finland and Russian populations (N, O, P, Q and V1, V2) are removed from the PCA, the unassigned populations are instead more closely related to populations of *F*. *vesiculosus* (Fig 2B). For both the strongly deviating SW Finland *F*. *vesiculosus* populations and the unassigned Gulf of Finland populations, it is noticeable that the populations are sampled over large to very large geographic areas and thus do not represent only a few isolated and aberrant populations (Fig 1).

Overall, these results show that geographic distances exceeding some hundred kilometres, may comprehensively affect the genetic structure of both *Fucus* species even inside the Baltic Sea. Indeed, genetic differentiation among the eastern and western Bothnian Sea populations of *F*. *vesiculosus* are of a similar magnitude or larger (*F*_ST_ 14–30% over 200–250 km, Table b in S2 Table) as differentiation among populations of *F*. *vesiculosus* over the North Sea—southern Baltic Sea transition zone (5–20% over 600–800 km [8]).

Such a remarked genetic divergence may have various explanations. One possibility is that part of this differentiation is due to bottlenecks during the colonization and expansion of the species' distribution in the northern and eastern Baltic Sea [44].

A second factor that may contribute to genetic divergence among populations is barriers to dispersal. Inside the Baltic Sea, the circulation of water is mainly counter-clockwise [45, 46]. Direct observations combined with biophysical modelling predict high connectivity among central Baltic populations for *F*. *vesiculosus* that has bladders that promote floating [46]. In contrast, our genetic data suggests that populations of *F*. *vesiculosus* on either side the Bothnian Sea are strongly isolated. On the other hand, oceanographic models show low levels of connectivity between northern and western Bothnian Sea and Estonian waters, which is supported by data on genetic differentiation ([9] and the present study). In the Gulf of Finland a strong outflow of surface water from the Neva River efficiently creates a barrier between the southern and the northern side of the Gulf [47]. This oceanographic barrier may explain the genetic divergence between the Russian and Finnish populations that are relatively close but located on either side of the river outflow.

Asexual reproduction in both species [17] adds to the genetic structuring of the Baltic *Fucus*. Without recombination, new genotypes will only be formed by the addition of new mutations—as observed in a few large, old and widespread clones of *F*. *radicans*, [19]. Thus in areas dominated by large clones, genetic structures will largely be preserved in the absence of sexual reproduction and genetic recombination.

Indeed, evolution slows down in areas where asexual recruitment is predominant and populations may, for example, take longer time to reach Hardy-Weinberg equilibrium [48], while in areas with high level of sexual recruitment there is a potential for more rapid evolutionary changes to occur. Asexual recruitment may in addition lead to a heavily skewed sex-ratio (observed in many of the *F*. *radicans* populations of N-W Bothnian Sea [19]) and in this way largely prevent not only sexual recruitment within species but also hybridization between the two species, which is common between other closely related fucoid species [3]. If hybridization occurs, it may be more important in Estonia and Gulf of Finland due to the high level of sexual recruitment in this area (but see [49] for potential phenological barriers to hybridization in Estonia).

The finding of relatively high levels of sexually recruited individuals in the inner parts of Gulf of Finland where salinity is only 2–3 PSU was unexpected, as earlier studies have argued that sexual reproduction is impeded in salinities below ~5 PSU due to polyspermy and subsequent failure of embryo development [22, 50]. Occasional upwelling of more saline waters [25] may temporarily allow sexual fertilisation and successful sexual recruitment in the innermost parts of the Gulf of Finland. A very speculative alternative is that increased levels of osmolality from the nutrient-rich River Neva may enable a higher rate of sexual recruitment in the Gulf of Finland than in other low-saline localities.

Alternatively, the distribution of asexual and sexual activity in the Baltic Sea fucoids, in general, may be a stochastic process following a recent colonization and spread into a new area by species capable of uniparental recruitment [51, 52].

In conclusion, the population genetic structure of the Baltic Sea *Fucus* populations is complex and illustrates what happens when divergence within a species is strong enough to muddle separation to a closely related species. Earlier evidence of high rates of speciation in the *Fucus*, lineage suggests that this lineage is generally prone to local adaptation and ecological speciation [43]. The very recent colonization of the ecologically marginal marine Baltic Sea ecosystem may have stretched the potential of evolutionary divergence in *Fucus* to an extreme level. As illustrated here, such a situation with rapid evolution of two recently diverged lineages may promote genetic structures that challenge the classical dichotomy of traditional systematics.

## Supporting Information

S1 Table Per locus and population heterozygosity (genets only). The *F*_IS_ calculated according to Weir and Cockerham [33]. Significantly positive values (bold) indicate heterozygote deficiency, and significantly negative values (italic) indicate heterozygote excess after control of the false discovery rate for multiple testing following [34].
(DOCX)

S2 Table Genetic differentiation (*F*_*ST*_) between pairs of *Fucus* populations in the Baltic Sea. (a) *F*_*ST*_ between all 14 *F*. *radicans* populations. (b) *F*_*ST*_ values between all 12 *F*. *vesiculosus* populations. The four unassigned populations (R, S, T, U) are included in both matrices. Bold figure indicates *F*_*ST*_ estimate is significant after Bonferroni correction.
(DOCX)
