# Supplementary material for: Divergence within and among Seaweed Siblings (Fucus vesiculosus and F. radicans) in the Baltic Sea
Source: PLoS One. 2016 Aug 15;11(8):e0161266. doi: 10.1371/journal.pone.0161266 (PMC4985153; doi:10.1371/journal.pone.0161266)
Supplement: S1 Table — The FIS calculated according to Weir and Cockerham [33]. Significantly positive values (bold) indicate heterozygote deficiency, and significantly negative values (italic) indicate heterozygote excess after control of the false discovery rate for multiple testing following [34]. (DOCX) [file pone.0161266.s001.docx]

| **S1 Table. Per locus and population heterozygosity (genets only).** |  |  |  |  |  |  |
| --- | --- | --- | --- | --- | --- | --- |
| The *F*_IS_ calculated according to Weir and Cockerham [33]. Significantly positive values (bold) indicate heterozygote deficiency, and significantly negative values (italic) indicate heterozygote excess after control of the false discovery rate for multiple testing following [34]. | | | | | | |
|  |  |  |  |  |  |  |

|  | Population | Locus | | | | | | | | | Number of loci with | |
| --- | --- | --- | --- | --- | --- | --- | --- | --- | --- | --- | --- | --- |
| Species |  | Fsp1 | Fsp2 | Fsp3 | Fsp4 | L20 | L38 | L58 | L85 | L94 | het. defic. | het. excess |
| *F. vesiculosus* | A | 0.16 | **0.54** | **0.44** | -0.11 | **0.24** | 0.13 | -0.14 | 0.13 | 0.21 | 3 | 0 |
| *F. vesiculosus* | B | 0.02 | **0.51** | **0.27** | -0.02 | 0.26 | -0.21 | 0.00 | 0.04 | 0.05 | 2 | 0 |
| *F. radicans* | C | 0.66 | *-0.47* | -0.67 | -0.38 | *-0.19* | -0.55 | -0.52 | *-0.85* | 1.00 | 0 | 3 |
| *F. radicans* | D1 | **1.00** | *-0.55* | -0.58 | -0.48 | *-0.54* | *-0.22* | -0.19 | -0.46 | -0.07 | 1 | 3 |
| *F. vesiculosus* | D2 | **0.21** | **0.23** | -0.03 | *-0.26* | **0.03** | **0.08** | -0.23 | *-0.66* | -0.17 | 4 | 2 |
| *F. radicans* | E1 | 1.00 | -1.00 | -1.00 | -1.00 | -1.00 | -1.00 | -1.00 | -1.00 |  | 0 | 0 |
| *F. vesiculosus* | E2 | -0.17 | *-0.65* | 0.19 | *-0.69* | **1.00** | *-0.27* | -0.60 | 0.12 | 0.59 | 1 | 3 |
| *F. radicans* | F1 |  | -0.14 | -0.60 | -0.14 | -0.14 | -0.28 | -0.60 | -0.33 |  | 0 | 0 |
| *F. vesiculosus* | F2 | 0.01 | 0.26 | **0.69** | 0.12 | 0.08 | 0.16 | -0.10 | -0.38 | 0.09 | 1 | 0 |
| *F. radicans* | G |  | -0.32 | 0.06 | -0.60 | -0.45 | -0.33 | -0.33 | -0.45 |  | 0 | 0 |
| *F. radicans* | H | -0.09 | -0.33 | 0.14 | -0.44 | -0.09 | -0.20 | -0.09 | -0.33 |  | 0 | 0 |
| *F. radicans* | I | -0.17 | -0.58 | -0.56 | *-0.78* | -0.40 | -0.56 | -0.40 | -1.00 |  | 0 | 1 |
| *F. radicans* | J | -0.05 | -0.19 | 0.29 | -0.20 | -0.10 | 0.12 | -0.29 | -0.39 |  | 0 | 0 |
| *F. radicans* | K |  | -0.10 | 0.00 | -0.20 | 0.36 | -0.20 | -0.39 | -0.67 | 1.00 | 0 | 0 |
| *F. radicans* | L | 0.00 | 0.18 | **0.43** | -0.07 | 0.12 | 0.05 | -0.09 | -0.16 | -0.21 | 1 | 0 |
| *F. radicans* | M | 0.05 | 0.08 | **0.50** | 0.22 | 0.18 | -0.06 | -0.13 | -0.26 | -0.08 | 1 | 0 |
| *F. vesiculosus* | N | -0.09 | -0.23 | 0.05 | -0.18 | 0.31 | -0.09 | -0.07 | -0.30 |  | 0 | 0 |
| *F. vesiculosus* | O | -0.23 | **0.30** | -0.05 | -0.13 | 0.04 | 0.10 | 0.15 | 0.31 | -0.13 | 1 | 0 |
| *F. vesiculosus* | P | **0.08** | **0.35** | -0.10 | 0.00 | **0.01** | -0.15 | 0.21 | -0.01 | 0.19 | 3 | 0 |
| *F. vesiculosus* | Q | 0.01 | **0.25** | -0.10 | -0.02 | 0.11 | -0.09 | -0.02 | -0.12 | -0.03 | 1 | 0 |
| Unassigned | R | 0.08 | **0.48** | **0.26** | -0.34 | **0.25** | -0.27 | -0.06 | *-0.37* | -0.04 | 3 | 1 |
| Unassigned | S | -0.39 | **0.68** | **0.29** | 0.08 | -0.09 | -0.32 | -0.06 | -0.38 | -0.33 | 2 | 0 |
| Unassigned | T | 0.06 | **0.60** | 0.04 | -0.20 | **0.25** | -0.08 | -0.08 | *-0.05* | 0.26 | 2 | 1 |
| Unassigned | U | 0.01 | **0.69** | **0.34** | 0.11 | -0.02 | -0.12 | -0.12 | 0.03 | 0.32 | 2 | 0 |
| *F. radicans* | V1 | 0.25 | 0.08 | 0.19 | -0.16 | -0.06 | **0.06** | 0.26 | -0.08 | 0.22 | 1 | 0 |
| *F. vesiculosus* | V2 | -0.47 | 0.05 | -0.47 | -0.14 | *-0.42* | -0.18 | 0.06 | *-0.91* | 0.00 | 0 | 2 |
| *F. radicans* | W1 | **0.13** | **0.44** | **0.23** | 0.36 | **0.56** | 0.26 | 0.11 | **0.49** | 0.45 | 5 | 0 |
| *F. vesiculosus* | W2 | **0.84** | 0.47 | 0.52 | **0.83** | **0.52** | 0.38 | 0.63 | 0.58 |  | 3 | 0 |
| *F. radicans* | X | 0.34 | **0.30** | 0.06 | 0.27 | **0.54** | **0.30** | -0.06 | **0.31** | 0.48 | 4 | 0 |
| *F. vesiculosus* | Y | 0.32 | **0.76** | **0.40** | 0.22 | **0.54** | 0.02 | 0.14 | 0.16 | -0.18 | 3 | 0 |
| Populations with het. deficiency | | 5 | 13 | 10 | 1 | 10 | 3 | 0 | 2 | 0 |  |  |
| Populations with het. excess | | 0 | 3 | 0 | 3 | 3 | 2 | 0 | 5 | 0 |  |  |
