# Supplementary material for: Divergence within and among Seaweed Siblings (Fucus vesiculosus and F. radicans) in the Baltic Sea
Source: PLoS One. 2016 Aug 15;11(8):e0161266. doi: 10.1371/journal.pone.0161266 (PMC4985153; doi:10.1371/journal.pone.0161266)
Supplement: S2 Table — (a) FST between all 14 F. radicans populations. (b) FST values between all 12 F. vesiculosus populations. The four unassigned populations (R, S, T, U) are included in both matrices. Bold figure indicates FST estimate is significant after Bonferroni correction. (DOCX) [file pone.0161266.s002.docx]

**S2 Table. Genetic differentiation (*F_ST_*) between pairs of *Fucus* populations in the Baltic Sea.**

(a) *F_ST_* between all 14 *F. radicans* populations. (b) *F_ST_* values between all 12 *F. vesiculosus* populations. The four unassigned populations (R, S, T, U) are included in both matrices. Bold figure indicates *F_ST_* estimate is significant after Bonferroni correction.

| **a)** |  |  |  |  |  |  |  |  |  |  |  |  |  |  |  |  |  |
| --- | --- | --- | --- | --- | --- | --- | --- | --- | --- | --- | --- | --- | --- | --- | --- | --- | --- |
|  | C | D1 | E1 | F1 | G | H | I | J | K | L | M | R | S | T | U | V1 | W1 |
| D1 | **0.03** |  |  |  |  |  |  |  |  |  |  |  |  |  |  |  |  |
| E1 | **0.06** | 0.09 |  |  |  |  |  |  |  |  |  |  |  |  |  |  |  |
| F1 | **0.01** | **0.02** | **0.05** |  |  |  |  |  |  |  |  |  |  |  |  |  |  |
| G | **0.06** | **0.05** | **0.17** | **0.07** |  |  |  |  |  |  |  |  |  |  |  |  |  |
| H | **0.12** | **0.09** | **0.18** | **0.12** | **0.02** |  |  |  |  |  |  |  |  |  |  |  |  |
| I | 0.04 | **0.04** | **0.11** | **0.04** | **0.02** | **0.01** |  |  |  |  |  |  |  |  |  |  |  |
| J | 0.13 | **0.09** | **0.21** | **0.13** | **0.00** | 0.00 | **0.06** |  |  |  |  |  |  |  |  |  |  |
| K | **0.06** | **0.02** | **0.11** | **0.02** | 0.01 | 0.02 | **0.04** | **0.00** |  |  |  |  |  |  |  |  |  |
| L | **0.09** | **0.11** | 0.12 | **0.05** | **0.11** | 0.08 | **0.07** | 0.12 | **0.04** |  |  |  |  |  |  |  |  |
| M | **0.11** | **0.13** | **0.15** | **0.08** | **0.14** | **0.13** | 0.12 | 0.16 | **0.05** | 0.01 |  |  |  |  |  |  |  |
| R | **0.13** | **0.14** | **0.14** | **0.10** | **0.18** | **0.16** | **0.14** | **0.23** | 0.11 | **0.14** | **0.14** |  |  |  |  |  |  |
| S | **0.18** | **0.21** | **0.17** | **0.16** | **0.26** | **0.23** | **0.20** | **0.32** | **0.19** | 0.16 | 0.16 | 0.02 |  |  |  |  |  |
| T | **0.17** | **0.20** | 0.16 | **0.15** | **0.22** | **0.21** | **0.18** | 0.27 | **0.17** | **0.20** | **0.20** | 0.04 | 0.05 |  |  |  |  |
| U | **0.15** | **0.18** | 0.13 | 0.12 | **0.21** | **0.20** | **0.17** | **0.27** | 0.15 | 0.17 | **0.17** | **0.03** | **0.03** | 0.01 |  |  |  |
| V1 | **0.25** | **0.28** | **0.17** | **0.23** | **0.32** | **0.31** | **0.28** | **0.35** | **0.24** | 0.23 | **0.23** | **0.17** | **0.16** | 0.17 | **0.16** |  |  |
| W1 | **0.22** | **0.21** | **0.17** | **0.18** | **0.27** | **0.26** | **0.24** | **0.31** | 0.17 | **0.22** | **0.20** | **0.08** | **0.11** | **0.09** | **0.08** | **0.16** |  |
| X | **0.24** | **0.23** | **0.20** | **0.21** | **0.29** | **0.28** | **0.26** | **0.32** | 0.20 | **0.23** | **0.21** | **0.10** | **0.13** | **0.11** | **0.11** | **0.16** | **0.01** |

| **b)** |  |  |  |  |  |  |  |  |  |  |  |  |  |  |  |
| --- | --- | --- | --- | --- | --- | --- | --- | --- | --- | --- | --- | --- | --- | --- | --- |
|  | A | B | D2 | E2 | F2 | N | O | P | Q | R | S | T | U | V2 | W2 |
| B | **0.09** |  |  |  |  |  |  |  |  |  |  |  |  |  |  |
| D2 | **0.13** | 0.16 |  |  |  |  |  |  |  |  |  |  |  |  |  |
| E2 | **0.11** | **0.18** | **0.11** |  |  |  |  |  |  |  |  |  |  |  |  |
| F2 | **0.12** | **0.17** | **0.18** | **0.27** |  |  |  |  |  |  |  |  |  |  |  |
| N | 0.21 | **0.28** | **0.23** | **0.25** | **0.30** |  |  |  |  |  |  |  |  |  |  |
| O | **0.11** | **0.18** | **0.18** | **0.16** | **0.17** | **0.07** |  |  |  |  |  |  |  |  |  |
| P | **0.11** | **0.14** | **0.14** | **0.15** | **0.15** | **0.08** | **0.01** |  |  |  |  |  |  |  |  |
| Q | **0.15** | **0.21** | **0.19** | **0.19** | **0.18** | **0.07** | **0.01** | **0.02** |  |  |  |  |  |  |  |
| R | **0.07** | **0.12** | **0.14** | **0.19** | **0.15** | **0.27** | **0.19** | **0.17** | **0.22** |  |  |  |  |  |  |
| S | **0.08** | **0.14** | **0.16** | **0.24** | **0.15** | **0.31** | **0.20** | **0.19** | **0.23** | **0.02** |  |  |  |  |  |
| T | **0.11** | **0.14** | **0.18** | **0.23** | **0.20** | **0.30** | **0.23** | **0.21** | **0.25** | **0.04** | **0.05** |  |  |  |  |
| U | 0.09 | **0.13** | **0.15** | **0.21** | **0.16** | **0.28** | **0.20** | **0.18** | **0.22** | **0.03** | **0.03** | **0.01** |  |  |  |
| V2 | **0.18** | **0.17** | **0.21** | **0.26** | **0.23** | **0.26** | **0.15** | **0.14** | **0.17** | **0.23** | **0.23** | **0.23** | **0.23** |  |  |
| W2 | **0.13** | 0.21 | **0.14** | **0.17** | **0.24** | **0.22** | **0.19** | **0.18** | **0.21** | **0.15** | **0.16** | **0.16** | **0.14** | **0.27** |  |
| Y | **0.06** | **0.14** | **0.17** | **0.13** | **0.21** | **0.24** | **0.16** | **0.17** | **0.22** | **0.10** | **0.14** | **0.11** | **0.11** | **0.23** | **0.09** |
